# What research means to the academic surgeon in the emerging era

**DOI:** 10.1002/ags3.12650

**Published:** 2022-12-26

**Authors:** Yuko Kitagawa

**Affiliations:** ^1^ School of Medicine Keio University Tokyo Japan

Surgical care has become increasingly segmented and sophisticated in recent years, requiring surgeons to stay abreast of an ever‐growing body of technology and knowledge. Meanwhile, the roles and importance of multidisciplinary therapy have expanded in surgical oncology. Opportunities for treatment through surgery alone have become limited, and the mainstream approach to oncological care incorporates perioperative therapy. Consequently, the surgical oncologist needs to possess advanced techniques and the ability to select the optimal surgical approach based on the preoperative assessment of local and systemic changes in the patient's condition. We are witnessing an ongoing evolution in nonsurgical approaches to comparatively early cancer treatment—endoscopic, local, chemoradio, and other therapies. Far‐advanced cancers that were unresectable with the established surgical therapy have become resectable through novel nonsurgical techniques. There are now more opportunities for so‐called surgical conversion, and with them more opportunities that require extremely challenging surgical procedures. With the recent addition of immune checkpoint inhibitors to the menu of options for perioperative care, caregivers must also consider autoimmune response‐related adverse events rarely encountered before. These and other changes have made surgery the last line of defense in certain areas. This naturally entails long periods of training, and it is becoming more challenging for young surgeons to balance research with their caregiving responsibilities.

Against this backdrop, debate continues to swirl around the question of the significance of basic research to the surgeon. In recent years, there have been cases where surgery has been transformed by clinical oncology translational research and by organ regeneration research applying regenerative medicine technology. And, the growth of research on diverse biomarkers is essential to the individualization of multidisciplinary therapy involving surgery. Going forward, academic surgeons and basic researchers must work closely together to advance research that connects the basic with clinical insights garnered from detailed clinical data and histopathological examination. In order to nurture academic surgeons capable of fulfilling this role, it is imperative that they be given opportunities to experience basic research at a relatively young age. At the same time, however, it needs to be remembered that academic surgeons are first and foremost surgeons, and accordingly their development must constantly rest on a foundation of surgical training.

While the situation varies from country to country, there is a general tendency for clinical research toward an academic degree to be considered less important than basic research. However, there are critical clinical questions that emerge from the clinic, and it is very important that they be addressed through clinical research using proper methods. For this reason, it is crucial for academic surgeons to master the skills needed to properly design randomized controlled studies and soundly carry them out, including with respect to ethical obligations. Moreover, big data‐driven research, which has become increasingly important in recent years, depends on the analytical skills needed to efficiently find answers in the real‐world clinic to the clinical questions raised. This comes at a time when the advancement of data science and the emergence of supercomputers have made it possible to rapidly process data in volumes much larger than could be handled before.

Today, surgical science is becoming more sophisticated and the need for collaboration with other domains is expanding—meaning that surgeons must become even more research‐minded. The *Annals of Gastroenterological Surgery*, as a platform for research by such surgeons, is committed to disseminating information to the world's gastroenterological surgery community.

## CONFLICT OF INTEREST

The author declares no conflicts of interest for this article.

